# Community engagement in deprived neighbourhoods during the COVID-19 crisis: perspectives for more resilient and healthier communities

**DOI:** 10.1093/heapro/daab098

**Published:** 2021-07-23

**Authors:** Lea den Broeder, Jane South, Auke Rothoff, Anne-Marie Bagnall, Firoez Azarhoosh, Gina van der Linden, Meena Bharadwa, Annemarie Wagemakers

**Affiliations:** 1 Faculty of Health, ACHIEVE, Amsterdam University of Applied Sciences, Tafelbergweg 51, Amsterdam 1105 BD, The Netherlands; 2 National Institute for Public Health and the Environment, PO Box 1, Bilthoven 3570 BA, The Netherlands; 3 Centre for Health Promotion Research, School of Health and Community Studies, Leeds Beckett University, Leeds LS1 3HE, UK; 4 Health and Society, Social Sciences Group, Wageningen University & Research, PO Box 8130, Wageningen 6700 EW, The Netherlands; 5 Indische Buurtbalie, Buurtruimte de Meevaart, Balistraat 48A, Amsterdam 1094 JN, The Netherlands; 6 St Eigenwijks, Albardakade 5-7, Amsterdam 1067 DD, The Netherlands; 7 Locality, 33 Corsham Street, London N1 6DR, UK

**Keywords:** community resilience, health promotion, community engagement, COVID-19

## Abstract

The current COVID-19 pandemic confines people to their homes, disrupting the fragile social fabric of deprived neighbourhoods and citizen’s participation options. In deprived neighbourhoods, community engagement is central in building community resilience, an important resource for health and a prerequisite for effective health promotion programmes. It provides access to vulnerable groups and helps understand experiences, assets, needs and problems of citizens. Most importantly, community activities, including social support, primary care or improving urban space, enhance health through empowerment, strengthened social networks, mutual respect and providing a sense of purpose and meaning. In the context of inequalities associated with COVID-19, these aspects are crucial for citizens of deprived neighbourhoods who often feel their needs and priorities are ignored. In this perspectives paper, illustrated by a varied overview of community actions in the UK and The Netherlands, we demonstrate how citizens, communities and organizations may build resilience and community power. Based on in-depth discussion among the authors we distilled six features of community actions: increase in mutual aid and neighbourhood ties, the central role of community-based organizations (CBOs), changing patterns of volunteering, use of digital media and health promotion opportunities. We argue that in order to enable and sustain resilient and confident, ‘disaster-proof’, communities, areas which merit investment include supporting active citizens, new (digital) ways of community engagement, transforming formal organizations, alignment with the (local) context and applying knowledge in the field of health promotion in new ways, focussing on learning and co-creation with citizen initiatives.

## Lay Summary

The COVID-19 pandemic is having a huge impact on community life and health, especially in poorer neighbourhoods. Many social problems have been made worse; e.g. poverty, difficulties getting food and social isolation. This has driven a huge effort from community-based organizations, national charities, volunteers and citizens who have worked hard to overcome these problems and help people in need. This paper shows examples of what has been happening in the UK and The Netherlands and how people quickly built on existing community work and made new projects in response to the pandemic. Activities ranged from local groups forming to help their neighbours through to national volunteer schemes. We argue that community action has been very important in meeting urgent needs, but also provides a base for future actions to make communities stronger and tackle underlying inequalities. Health promotion principles suggest that action is needed across society to give more power to communities and make sure that the priorities of disadvantaged communities are taken seriously in building a recovery. Practical actions include forming long-term partnerships between public services and community-based organizations, giving practical support to volunteers, making sure new digital-ways of connecting are open to everyone, and involving communities in doing research.

## INTRODUCTION

**Fig. 1:**
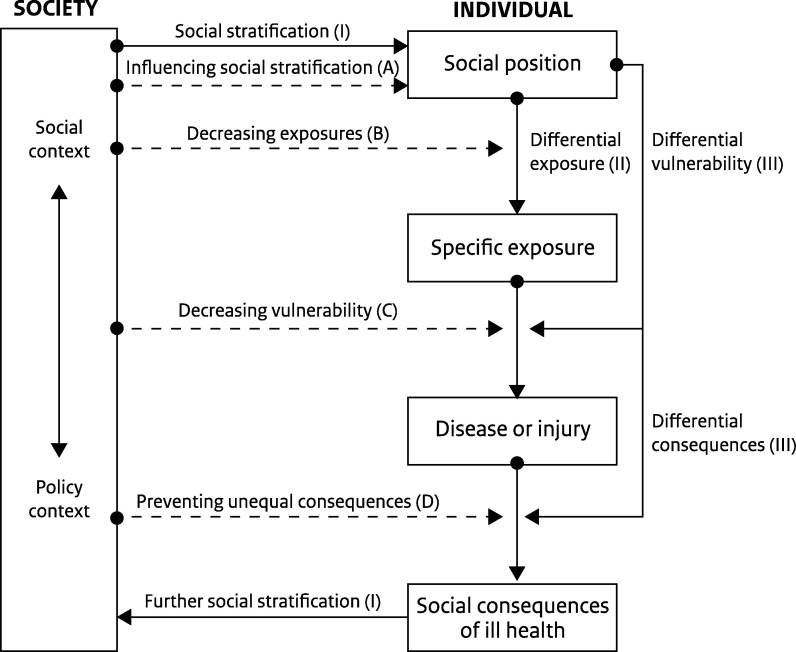
Impact of social inequalities on health outcomes. Source: Diderichsen *et al.* (Diderichsen *et al.*, 2001).

The current corona pandemic is a sudden, unexpected and extreme change that impacts organizations, citizens and communities. It demonstrated lack of preparedness for what a global pandemic would be like, how it would affect daily life, and the urgent need to deal with health threats and uncertainties. Inequalities exist in COVID-19 morbidity and mortality rates in Spain, USA and UK reflecting unequal experiences of chronic diseases and the social determinants of health (Bambra *et al.*, 2020). People in deprived communities in England and Wales are twice as likely to die compared to those living in non-deprived communities (O’Dowd, 2020) and run a higher risk of hospitalization with COVID-19 (Verhagen *et al.*, 2020). A similar pattern seems to exist in the Netherlands where morbidity and mortality are higher among those with pre-existing chronic diseases, several of which (obesity, coronary heart diseases and diabetes) are more common in people with a low socioeconomic position (https://www.rivm.nl/coronavirus-covid-19/risicogroepen). Citizens have a greater likelihood of infection when they work in essential services; have incomes near the poverty line; have fewer resources to stockpile food and heightened vulnerability to adverse effects of the virus once exposed (Schulz *et al.*, 2020). Health and economic impacts are positively correlated (Allen and Mirsaeidi, 2020) and adverse effects from pandemic containment measures, including financial insecurity, loss of job or livelihood, social isolation, increased risk of gender-based domestic violence (Douglas *et al.*, 2020; Polizzi *et al.*, 2020; Stellinga *et al.*, 2020; Usher *et al.*, 2020), are unequally distributed (Bambra *et al.*, 2020; Schulz *et al.*, 2020). A Public Health England review (Public Health England, 2020) found that significant disparities exist for Black, Asian and Minority Ethnic (BAME) communities in relation to COVID-19 and that long term disadvantage and discrimination have played a part. In the Netherlands, the high-level Working Group on the Social Impact of the Corona Crisis indicates that there is a severe social impact of the corona crisis in deprived areas because it enlarges existing problems in such areas regarding education, safety, health and poverty (Werkgroep Sociale Impact van de Coronacrisis, 2020). This is in line with the layered character of the impact of social inequalities on health outcomes as described by Diderichsen *et al.* (Diderichsen *et al.*, 2001) (see Figure 1). Therefore, responses to the pandemic should apply an equity lens: giving attention to the most vulnerable groups (Van den Broucke, 2020) and preferably through building action in the communities where they live. Community resilience is key in coping with catastrophic events (Coles and Buckle, 2004) like the COVID-19 pandemic. The World Health Organization (Ziglio *et al.*, 2017) proposes resilience operating at three levels—individual, community and across a system, and having four capacities: Adaptive (ability to adjust to disturbances and shocks), Absorptive (ability to manage and recover from adverse conditions using available assets), Anticipatory (ability to reduce disturbance and shocks by proactive action to minimize vulnerability) and Transformative (ability to develop systems better suited to change, uncertainty and new conditions) (Ziglio *et al.*, 2017; Thomas *et al.*, 2020). For this paper we merged definitions of community resilience addressing change and including the ability of communities to prepare, manage and learn (Wilson, 2013; Rippon *et al.*, 2020; Thomas *et al.*, 2020): ‘The capacity of a community to absorb disturbance, respond to and influence change, sustain and renew the community, develop new trajectories for the future, and learn so they can thrive in a changing environment’. In addition, we acknowledge that inclusive engagement of citizens and organizations, through a whole-of-society approach, is critical for a community's adaptive capacity to respond to adverse events (O’Sullivan *et al.*, 2015). Community engagement in change processes (Coles and Buckle, 2004) is an essential element in building resilient and healthy communities. For individuals facing (extreme) change, finding ways to engage during mass traumas is a robust predictor of increased psychological well-being (Polizzi *et al.*, 2020).

Community engagement is an action area of the Ottawa Charter for Health Promotion (World Health Organization, 1986), including social support, primary care, or improving urban space. Involving communities enhances health through empowerment, strengthened social networks, mutual respect, providing a sense of purpose and meaning (Kawachi and Berkman, 2000; Wagemakers *et al.*, 2010; Laverack and Keshavarz Mohammadi, 2011; O’Mara-Eves *et al.*, 2015; Bagnall *et al.*, 2019). In this paper, we focus on deprived neighbourhoods. Deprived communities face a multitude of problems in terms of poverty, poor housing and liveability, employment, and health. If a community, neighbourhood, or area, is deemed deprived or not is arbitrary; it is common to refer to the *level* of deprivation. Various countries use different, but overlapping, indicators to asses this deprivation level. The English Indices of Deprivation 2019 (IoD2019) (Ministry of Housing, Communities & Local Government, 2019), e.g. includes indicators on income, education, employment, health, crime, barriers to housing services and living environment. In the Netherlands, the 40 most deprived, or ‘priority’ neighbourhoods have been selected based on indicators in four category clusters: socioeconomic deprivation of households, resident-reported (social) liveability problems, physical deprivation regarding housing and resident-reported problems in the physical environment (Platform 31, s.a.). Community engagement has long been recognized as an important resource for health and a prerequisite for effective health promotion programmes in such neighbourhoods. In addition, it provides access to and for vulnerable groups and helps build insight into the assets and needs of residents. Notwithstanding the benefits of participation, becoming and remaining engaged in community activities can be more difficult for citizens in deprived neighbourhoods than elsewhere, due to the adverse impacts of socioeconomic factors and marginalization (UCL Institute of Equity, 2013).

Despite the severe economic, social and health consequences of the current crisis, the current situation has stimulated an extraordinary response from citizens: in self-organized groups or as individuals, through community-based organizations (CBOs) and by cooperating with formal (national and local) organizations. This has led to heightened awareness of ‘community’ in public health (Kluge, 2020; Yardley *et al.*, 2020). If we are to learn from this and translate learning into responsive and empowering community-based approaches for recovery, we need to develop analyses of what is occurring and how community action and community resilience can be strengthened. This means understanding health assets and vulnerabilities within communities (South *et al.*, 2018).

Therefore, the core question of this paper is: what lessons can be learnt through the current crisis and how can these be put into practice in new and responsive approaches in future? We present here a theoretical perspective on the potential of community action by citizens, CBOs and formal organizations in the UK and The Netherlands, with a focus on developing post-COVID-19 community-based recovery processes and utilizing methods building on neighbourhood assets. Such action may become a steppingstone towards building community resilience in deprived neighbourhoods. We support our argument by providing a structured list of real-life examples, as these illustrate salient features of community action during the pandemic.

## COVID-19 CRISIS, COMMUNITY ACTION AND EMPOWERMENT

In both the UK and The Netherlands, citizens, CBOs and formal organizations are playing a major role in meeting the high levels of health, social and economic need created by the COVID-19 crisis and building towards more resilient communities (Alakeson and Brett, 2020; https://wijamsterdam.nl; Locality, 2020; Reddish, 2020; Theunissen, 2020). In the UK, Alakeson and Brett state that ‘The Covid-19 crisis has been characterized by an extraordinary wave of social solidarity and community action sweeping across the country’ [(Alakeson and Brett, 2020), p. 2]. In The Netherlands, the Red Cross welcomed 30 000 new volunteers (Engbersen *et al.*, 2020; Movisie, 2020). To understand what happened and what lessons can be learned, we need to recognize the context for many deprived communities; one of widening socioeconomic and health inequalities (as outlined above). Many communities are facing poverty and disruption of income and food (European Foodbanks Federation, 2020). For The Netherlands, it has been calculated that poverty will increase by 25% until 2035 when policy remains unchanged; even without taking the impact of the COVID-19 crisis into account (CPB Economic Policy Analysis, 2020). Community action is adapting to this new context, addressing high levels of need in some communities and coping with social restrictions that caused neighbourhood activities to abruptly stop in March 2020 and meeting places closed.

In discussing the community response to the COVID-19 crisis and what support is needed for recovery, we present an overview of community action in the UK and The Netherlands (Table 1). We collected examples of community activities through our personal networks in communities, information gained by newsletters, reports and websites of healthcare and welfare organizations. Thus, we created a varied overview of different activities that have evolved during the outbreak, acknowledging that this is not comprehensive. In particular, it is important to note that many citizen initiatives go undocumented—such initiatives often remain unseen as they can only be identified by close investigation of local contexts. Recognizing the value of community practice, we had four in-depth (online) group discussions on how to categorize the various examples and their features. In addition, we conducted three author interviews (with informed consent) on community resilience, which capture the perspectives of a resident, a community worker and a development manager of a network of CBOs. In these 30-min phone interviews, three questions were posed: (i) What happened to existing community initiatives when the corona measures were put into place? (ii) What new initiatives emerged; what effects did they have? (iii) How do you perceive the future for community initiatives in your area? The interviewees were sent the interview reports for member checking and co-operated to include these in the paper (Table 2). We have used these examples of activities and the three perspectives (one for each type of initiative) to distil features of citizen-led initiatives, CBO-led and formal organization-led strategies to mitigate impacts of the pandemic at local level. Central to our approach was health promotion: we drew on its wealth of knowledge on how to initiate and support community action and resilience in future.

**Table 1:** Examples of community activities in response to COVID-19 in the UK and the Netherlands

	UK	The Netherlands
**Community or citizen-led** *Community/citizen-led responses are characterized by mutual aid, informal volunteering and community organizing.*	*Colvestone Crescent:* Whatsapp group to identify people who had been in contact with a neighbour diagnosed with COVID-19, and to exchange items and information about local food and medicine availability (Alakeson and Brett, 2020) *Mutual Aid COVID-19:* Website run entirely by volunteers displaying over 2,900 local mutual aid groups (Covid-19 Mutual Aid UK, 2020). This is part of a network of mutual aid groups across the world. *Citizen-led support in Bristol:* People who self-identified (in a survey) as being involved in supporting their neighbours do a wide range of tasks including food shopping, dog walking, gardening, support around food availability, providing information, and broader support to ‘raise the mood’ (e.g., painting rainbows in windows).	*De Hagedoorn:* A residents’ business exploited by residents without a subsidy, with its own foundation that owns the property that can do welfare work for and with the neighbourhood from its own proceeds (Theunissen, 2020). *Just People Who Want to Help People [Gewoon Mensen Die Mensen Willen Helpen]*: Website initiated by four students on which people can offer and demand help related to COVID-19 (https://www.gewoonmensen.nl/faq). *One-and-a-half-meter bench [Anderhalvemeterbankje]*: For neighbours to be able to meet and interact with others at a safe distance, a local artist collective developed a bench that made this possible: (https://www.anderhalvemeterbank.nl/). *Iftar-meals:* Breaking the fasting together (iftar) is a really important part of the Ramadan. Residents made meals for people to enjoy and eat collectively from their own homes, thus being connected during this important month (personal communication Gina van der Linden).
**Mixed –organizations and communities** *These responses link the work of community-based organizations with social action by citizens. Neighbourhood or community-based organizations have a key role in coordinating local efforts and addressing disadvantage.*	*Anglers Rest:* Community pub and hub housing Helpful Bamford, a volunteer group offering support with shopping and phone calls (Alakeson and Brett, 2020). *Bevy:* Community-owned pub delivering 100 meals to vulnerable elderly normally attending the weekly lunch club. Very connected to the local community, can therefore ensure that they provide appropriate meals for residents (Alakeson and Brett, 2020). *Homebaked*: Community bakery that switched to baking 50-70 fresh loaves daily for the local foodbank and community centre and delivering frozen pies for income generation and meeting further community need (Alakeson and Brett, 2020). *Isolation Station Hastings:* New online television channel for bringing together local people (Alakeson and Brett, 2020). *The Annexe:* Providing food to the most vulnerable and isolated residents. Handed out Easter eggs to children in the neighbourhood (Alakeson and Brett, 2020).	*At home with DUMS [Thuis met DUMS]:* Providing 20 daily online music lessons via videocalls for people aged 70+ to combat their isolation because of the COVID-19 crisis. People are able to lend a professionally disinfected musical instrument from the project (https://dums.nl). *NeighbourhoodMeals [BuurtMaaltijden]:* Organization helping locals to get in touch with people in their neighbourhood for whom they can cook an extra meal. By providing them with a home-cooked meal, local people want to reach out to their neighbours to let them know that they are there for them (https://www.youtube.com/watch?v=kGiPcMDE5zo; https://www.buurtmaaltijden.nl). *We Amsterdam [Wij Amsterdam]:* Platform for citizens to support each other that started by welfare work (500+ initiatives) (https://wijamsterdam.nl)
**Organization-led** *These responses are often initiated by public service or voluntary sector organizations and volunteering is coordinated through formal platforms/systems. They may evolve to have strong community partnerships or alliances.*	*Community Support Volunteers:* Volunteers helping people unable to leave their home due to COVID-19. Via telephone befriending, they help them to stay connected with the outside world and receive essential products (e.g. medicines) (Volunteering Matters, 2020a). *Inspired Neighbourhoods:* Made their diabetes and mental health support services available online (Alakeson and Brett, 2020). *NHS Volunteer Responders:* Organization run by the NHS and supported by Royal Voluntary Service that offers support to people in need and to people who are avoiding public places (Royal Voluntary Service, 2020b).*Play Wales:* National charity providing information about active play in and around the home (https://mailchi.mp/8179d10e4843/hepa-europe-newsletter-may2020). *Remote Media Champion:* online resource including art, sport, music and well-being resources to help young people through the COVID-19 pandemic (Volunteering Matters, 2020b).	*Balcony Fit [Balkon Fit]:* Weekly activity whereby older or vulnerable people tied to their homes can exercise on their balconies together with a sports instructor (Sportservice Wageningen, 2020). *Coronahelpers:* Platform linking supply and demand for help during the COVID-19 crisis (https://www.coronahelpers.nl). *Eurus:* Developing several methods that keep in mind the social distancing measures, e.g. the ‘birdhouses method’, whereby birdhouses (or anything similar) are placed at strategic places and people know that they can get their information or ‘assignment’ there. Very useful when the opinion of neighbourhood residents is needed for a process (Theunissen, 2020). *Movisie:* Providing an online meeting about citizen participation with experts, a councillor, and an active resident (De Bruijn, 2020). *MyNeighbourhood [MijnBuurtje] & NeighbourhoodConnect [WijkConnect]:* Online neighbourhood platforms that have a special COVID-19 variant or that are temporarily providing their services for free (Bubic, 2020).

**Table 2:** Narratives about community action during COVID-19 crisis in the UK and the Netherlands

**Firoez Azarhoosh, active citizen, Amsterdam, the Netherlands (community-led initiative)**
When the corona crisis began and measures were taken, this immediately caused a lot of initiatives in our community to be placed on the backburner. The result was people with problems disappeared out of sight. As a group of active citizens, we started a project to hand out meals for those in need. This enabled us to get into contact with community members in complex situations. Their financial or societal position had been precarious all along, but due to the crisis, their last options to make ends meet were lost. I think mutual help in this crisis strengthened social cohesion. The challenge is now, to develop a sustainable strategy and long-term solutions for the problems of vulnerable people in our community. We need to develop professional coordination for our community action, but unlike community volunteers, existing formal organizations have not adapted to the new situation and their procedures remain the same—there is a misfit here. We see three important tasks for the future: (1) start a cocreation process with local authorities and professionals to develop new strategies; (2) leading to ways to protect and nourish the newly developed initiatives; (3) while focusing on the neighbourhood, not city level to ensure relevance and recognizability for our community.
**Gina van der Linden, community worker, Eigenwijks,^a^ Amsterdam, the Netherlands (mixed organization and community initiatives)**
Residents in our communities felt a strong need to help others during this period of crisis. As a community organization, we see it as our duty to support that. Residents know so much more about people in their community than professionals do, so it is our job to offer trust, support and help. We have to make sure not to take over or interfere with their activities. No control, no calling to account. Trust was the key word—and this was good for residents and professionals alike. Usual procedures for funding requests were widened. They were doing a stupendous job—offering neighbours and fellow residents aid in heart-breaking circumstances. We helped just by asking how they were doing and listening to their stories. Neighbourhood bonds became stronger, perhaps because people were confined to their nearby environment. We also saw people adapting by learning; digital literacy increased enormously for example. Of course, we also saw many difficulties related to poverty as the informal economy was hit hard, and much more difficulties are expected yet to come. I do hope the community power and cohesion as well as trust will remain.
**Meena Bharadwa, Development Manager, Locality, UK (organization-led initiatives)**
On reflection, the pace at which community organizations responded was astounding—although we shouldn’t be surprised. Many of Locality’s member organizations^b^ repurposed activities and developed new services rapidly—e.g. turning a food hub to a food parcel delivery service in 24 h. What I have noticed is where there is a local infrastructure and there has been investment in long term partnerships, community organizations have been able to respond quickly and effectively, delivering the right support at the right time to the right people. These organizations have acted as ‘cogs of connection’ between residents and services. Being trusted meant that they could rapidly mobilize support—one community organization in Birmingham ended up coordinating 800 local volunteers. But infrastructure is not equal and where that deep-rooted local intelligence hasn’t been built up, the response is more limited, and mutual aid groups have not got anything to connect to. It is shown again the importance of trust built up over time. And the need for a localized not a centralized system in order to get help to those in need. Priorities going forward. Firstly, we need to tackle the systemic, structural issues around the economy and make sure we have bottom-up growth that does not leave people behind and facing hardship. Communities should not be separate from economic growth. Secondly, we need services addressing the wider determinants, co-designed and led by communities. Communities have demonstrated how to deal with a crisis—so why not have that level of trust afterwards? Finally, thinking about the huge impact of COVID-19 on BAME communities where the loss of community members is being keenly felt. What can we do to support these communities better? So often BAME community groups have less funding, less support, less access to buildings etc. So going forward, we need to make sure support is targeted to these communities.

a Eigenwijks is the largest residents’ organization in Amsterdam Nieuw-West district. It supports residents in building socially strong, liveable communities.

b Locality is the national membership network for community organizations (UK). Their goal is to help community organizations to be the best they can be and to create a supportive environment for their work.

Tables 1 and 2 show the broad range and diversity of community responses in both the UK and The Netherlands. These reflect varying degrees of community ownership and formality and a responsiveness to disadvantage and vulnerability. This is in line with the observation that the ‘formal’ level of participation, e.g. according to Arnstein’s ladder (Arnstein, 1969) can be less important for the quality of the community’s engagement than the actual participation mechanisms and how they are experienced by community members (Tritter and McCallum, 2006; Cornwall, 2008). Based on our in-depth discussions and on health promotion literature, we have inductively grouped these into six features demonstrating how citizens, CBOs and formal organizations began to build resilience and community power.

- *Mutual aid as a key part of the response*. Across the world, there has been an increase in mutual aid groups, where citizens self-organize to support each other and those made vulnerable by the pandemic (Covid-19 Mutual Aid UK, 2020). Mutualism has always been a feature in deprived communities (Hardill *et al.*, 2007; Baldacchino *et al.*, 2008; Marks, 2012), and also a strong theme in the labour movement (Hobsbawm, 1984), but mutualism has not tended to feature strongly in the public health discourse. In this pandemic, strong citizen-led responses have been observed in both the UK and The Netherlands. Mutual aid and informal volunteering (defined as volunteering outside of an organizational context; Lee and Brudney, 2012) have often been the mechanisms to provide vital support in the most challenged communities (Alakeson and Brett, 2020).
- *Neighbourhood ties being the cornerstone of community action.* The importance of hyper-local activity, often street by street, appears to be a central feature of much of the neighbour-based community action in the pandemic. The evolving role of local associations and businesses, like bakeries and pubs, align to the principles of Asset Based Community Development (Kretzmann and McKnight, 1993; Blickem *et al.*, 2018). Local knowledge is critical in an outbreak and social networks help reach people who need support, a point also learnt in other outbreaks (Laverack and Manoncourt, 2016; Laverack, 2017).
- *The central role of CBOs in deprived neighbourhoods*. Community centres and hub organizations, which often run a mix of health promotion and social activities, are critical assets in many deprived neighbourhoods (Bertotti *et al.*, 2012; Bagnall *et al.*, 2018). In this pandemic, CBOs have acted as hubs, rapidly repurposing activities, coordinating volunteers and food supplies. Locality, a UK-wide network of CBOs, concluded that existence of local community infrastructures was critical in local response and that ‘the role of community organizations as ‘cogs of connection’ has been strengthened’ [(Locality, 2020), p. 7]. CBOs have changed quickly and larger organizations, including public services, relied on CBOs to reach those in need (Alakeson and Brett, 2020; Locality, 2020).
- *Changes in patterns of volunteering*. Significant volunteering responses have been seen in many European countries (Kluge, 2020). In the UK, there has been a reported growth of informal (Office for National Statistics, 2020) and formal volunteering (Reddish, 2020). In the UK, new national schemes developed, recruiting volunteers as part of the COVID-19 response, e.g. NHS Responder scheme (Royal Voluntary Service, 2020a,b), but these schemes relate to wider trends of neighbourliness underpinned by an essential solidarity with those in need (Office for National Statistics, 2020). The skills and experience of volunteer-involving organizations have been tested as existing volunteers have taken on new roles and new volunteers recruited and trained.
- *Use of digital media to connect people and toorganizeactivities*. Different and innovative digital media have been used to organize and deliver community action. Social media has been used to connect to people, provide e-mail or phone support for active volunteers, developing digital neighbourhood platforms/meeting points. In some cases, whole new online resources have been created to support collective activities.
- *Community activities are health promoting*. Participation in activities and social interaction is healthy in itself, next to, e.g. the benefits of being physically active as in the BalkonFit activity (Sportservice Wageningen, 2020). This shows the core values of equity, participation and empowerment of the WHO Ottawa Charter (WHO Ottawa Charter, 1986) in practice, a success that in many ‘regular’ health promotion programmes needs a lot of investment and takes a long time. Most community activities have been initiated to relieve the immediate consequences of the COVID-19 crisis, e.g. lack of contact, lack of resources for food, lack of physical activity; however, there are more possibilities for promoting health, e.g. activities directed at food provision might provide healthy food. In many cases, these actions reveal new needs and pathways to future approaches. Practices may thus change and develop under difficult circumstances, drawing on community power.

## DISCUSSION—LEARNING INTO PRACTICE

In this paper we addressed community resilience as ‘the capacity of a community to absorb disturbance, respond to and influence change, sustain and renew the community, develop new trajectories for the future, and learn so they can thrive in a changing environment’. In the UK and the Netherlands, we have seen many good examples of citizen-led, CBO-led and organizational responses to change, sustaining the community and absorbing disturbance.

The overall picture is that community action has grown rapidly in response to human need and a desire to contribute to society. This is like previous crisis situations. Lessons from the Ebola crisis suggest that community engagement was a critical factor in outbreak management (Laverack and Manoncourt, 2016). In the aftermath of 9/11, many people found meaning in the attacks and experienced increased sense of control, belonging and self-esteem by giving support to friends and family and the larger community (Peterson and Seligman, 2003). A similar process seems to be going on now. Current community actions in response to the COVID-19 crisis go beyond individual growth; they provide a solid basis for creating sustainable communities cooperating with public services on a basis of mutual respect and trust. This is particularly important in deprived communities as the level of neighbourhood social capital and resilience have an impact on the health of the residents (Bartley, 2011; Mohnen *et al.*, 2011). In addition, recent research in the Netherlands provides evidence that existing social capital mitigates the adverse impact of the COVID-19 crisis on mental health (Engbersen *et al.*, 2020). Wilson (Wilson, 2013) wrote about ‘social memory’ and social learning influencing community resilience pathways in the context of the Christchurch earthquakes. Ideally, resilience is not about bouncing back to the pre-shock state but about evolving into something better (Thomas *et al.*, 2020). In this, health promotion has a crucial role to play, by addressing upstream factors that contributed to excess impact of COVID-19 in deprived communities and that have a broader meaning and impact (Schulz *et al.*, 2020). Using the Ottawa Charter framework, including strengthening community action, can increase effectiveness of programmes (Fry and Zask, 2017) or as Van de Broucke has put it: ‘The models, strategies and case examples of successful community action and empowerment documented by health promotion researchers and practitioners over the years can provide guidance to communities facing the challenge of the COVID-19 pandemic.’ [(Van de Broucke, 2020), p. 4].

In order to support this process, we suggest that health promotors and policy makers need to work at different levels: with citizens, CBOs and formal organizations. Drawn from our reflections and analysis, we propose the following as areas that merit investment.

### Supporting active citizens

At the community level, working in partnership with local groups, CBOs and individuals offers health promotors a critical connection point during and after the pandemic. Citizens may be better equipped to tune in with the lives, challenges and priorities of fellow residents. Earlier research has shown the importance of volunteers, and other lay health workers, bringing their unique experiential knowledge and being able to bridge between services and marginalized or underserved groups [e.g. (South *et al.*, 2011, Wagemakers *et al.*, 2015; Den Broeder *et al.*, 2017)]. The extraordinary humanitarian efforts being made by some of most disadvantaged communities need to be recognized and approached with some cultural humility. In addition, while community action is an act of altruism, it needs practical support and is not cost-free (South *et al.*, 2014). Active citizens and CBOs may need funding to ensure out-of-pocket expenses are met quickly, training where new roles are taken, transport and finally, opportunities to link up with others. This should be done inter-sectorally by all partner organizations working in a neighbourhood. A limitation is that it is hard to find examples of citizen-led initiatives, as they are not linked to (formal) organizations nor publish their activities online or in newspapers. This means that many citizen activities go unseen by professionals, which we experienced as well in identifying citizen-led activities in Table 1. Overall, the contributions of active citizens and CBOs need to be recognized within the public health response and valued equally with professionally led volunteer schemes.

### Supporting new digital ways of community engagement

This pandemic brought about new approaches in community action. These new pathways need to be pursued. Volunteering may still be difficult for groups experiencing disadvantage and marginalization (Southby *et al.*, 2019) and learning and development of community capacity is key. Most importantly, there is an urgent need to enable digital engagement which has proved useful, and might contribute to digital literacy, but may not yet be an option for all. In the UK, 15.2 million people are estimated to be non-users of the internet in 2017 (Good Things Foundation, 2017), 8% (4.3 million people) to have zero basic digital skills and a further 12% (6.4 million adults) to have only limited online skills (Office for National Statistics, 2019). Although the population of the Netherlands is advanced in terms of digital skills, only 30% of the people with a low educational level have digital skills that exceed the most basic level. Of the 65- to 75-year-olds this rate is 18% (https://ec.europa.eu/eurostat/web/products-datasets/-/tepsr_sp410). But skills do not suffice to close the digital gap; the costs of connecting to the digital world should be reduced or compensated for, recognizing that this is a basic need for all in current society.

### Transforming organizations

Laverack argues that health promotion in disease outbreaks should go beyond community engagement to use empowerment approaches that foster community ownership and enable communities to develop local action and supportive social networks (Laverack, 2017). Much can be learnt from the collective wisdom of CBOs that relate to groups that face the worst inequalities in the COVID-19 pandemic. The role of CBOs is also critical to recovery and long-term investment is needed to ensure these organizations can continue to act as connection points for services and communities. There is a need to address the cultural and organizational barriers in systems and public services that often serve as barriers to participation and prevent community voices from being heard (Harden *et al.*, 2015). Transforming communities to become more resilient requires that public services also change their focus and operations. It is important that these services adapt to local experience, culture and history (Denters and Klok, 2010). The Public Health England review on COVID-19 inequalities for BAME groups (2020) highlights the need to build culturally competent prevention services and that ‘fully funded, sustained and meaningful approaches to tackling ethnic inequalities must be prioritised’ (p. 11). It is also important to recognize that the work field between citizens, CBOs and formal organizations is dynamic and sometimes ‘fuzzy’. Formal organizations’ activities may become strongly rooted in citizens’ daily lives and experienced as owned by them. Reversely, citizen initiatives may get institutionalized (Soares da Silva, 2018). Thus, transformation takes time and a complex learning process. Analysing other countries’ experiences provides useful lessons for policy and practice in implementing resilience-enhancing strategies.

### Role of the context

Local contexts are more important than ever, now that people depend heavily on their immediate environment. This requires taking such contexts into account. We cite Alakeson and Brett (Alakeson and Brett, 2020) who state that ‘The idea of “community” is still habitually seen in policy circles as a sideshow; as something which is nice to support and worth throwing little bits of money at, but never the answer to any of the big public policy questions of our time.’ (p. 4). The current crisis, and communities’ responses, may—and should—change that point of view. Context-sensitivity also means that whole system approaches and an understanding of the social determinants of health, core in health promotion, should be applied (Naaldenberg *et al.*, 2009; Kickbusch and Gleicher, 2012). Forming long-term alliances and trusting relationships in and with communities is key; they place communities and civil society organizations at the heart of decision making and action. Furthermore, possibilities for community action depend on local and national policies, e.g. social policies matter to crisis management and recovery, and the regime type matters as well as formal political institutions and a state’s capacity (Greer *et al.*, 2020). Therefore, various geographic regions should be studied, variation in context considered and explained.

### Knowledge agenda

New approaches and future trajectories need to be developed, based on learnings from the COVID-19 crisis and drawing from the rich body of knowledge in health promotion. This should include, as Schulz *et al.* (Schulz *et al.*, 2020) propose, authentic engagement of community voices in research and change processes, strategic use of scientific evidence to impact policy change, building skills and capacity of all partners to effect policy change, and developing multilevel and multisectoral interventions using rigorous evaluation methods, and applying non-disease-specific approaches that address structural conditions that impact health inequities. Tried and tested methods like CBPR may offer starting points to gain vital community insights and jointly explore solutions in this current crisis (Wallerstein and Duran, 2010; Public Health England, 2020), further informed by knowledge from community-based restoration after disasters in deprived communities (Denters and Klok, 2010).

Thus, in addition to focusing on how to address the problems caused by (the measures to stop) the spread of the coronavirus, we propose drafting a community resilience knowledge agenda for health promotion in and with deprived communities. A first and important step would be, to develop a conceptual framework and indicators to guide systematic collection of data on COVID-19 related community activity and their type of health promotion source in deprived areas. Because many resident-driven activities are difficult to identify, it is important to draw on local community knowledge: people living in an area can be valuable partners in finding and describing the activities. Our initial analysis of practice examples has highlighted the potential significance of community knowledge in understanding dynamic community-based responses to the pandemic. These data should be studied in-depth and analysed to better understand how the six features mentioned above contribute to the emergence, development and success/failure of these activities, and the impact on local communities’ health and wellbeing. In particular, it will be useful to compare between specific feature examples, and between geographical settings and regions. The knowledge agenda should be amended by new topics as they present themselves during coming times. Moreover, evidence-based approaches should be developed and tested that support community organizing and citizen-led action (Rippon *et al.*, 2020).

## CONCLUSION—TOWARDS RESILIENT COMMUNITIES

In this paper, we applied a health promotion lens identifying features of community action examples from the UK and The Netherlands and distilling six features of community actions: increase in mutual aid and neighbourhood ties, the central role of CBOs, changes in volunteering and use of digital media and health promotion opportunities. Based on that, we reflected on how this community action can be (further) enabled and supported, particularly in deprived areas where there are major inequalities or where civil society infrastructure is weak, and what is needed for this transformative change to happen. We argue that in order to enable and sustain resilient and confident, ‘disaster-proof’ communities, areas which merit investment include supporting active citizens, new (digital) ways of community engagement, transforming formal organizations, alignment with the (local) context and applying health promotion knowledge in new ways, focussing on learning and co-creation with citizen-led initiatives. A robust knowledge agenda, yielding research that informs policy and practice is one key element. To build stronger, more resilient and more inclusive communities, we need to give focus and support to neighbourhoods and communities, which face the worst inequalities and work alongside active citizens and CBOs in those communities to co-create knowledge, strengthen supportive networks and meet health and social needs.

## ETHICAL APPROVAL

The three author interviews reported in this paper were conducted with informed consent of the interviewees. In addition, the interviewees cooperated in writing this paper.
